# Information Flow Analysis between EPU and Other Financial Time Series

**DOI:** 10.3390/e22060683

**Published:** 2020-06-18

**Authors:** Can-Zhong Yao

**Affiliations:** School of Economics and Commerce, South China University of Technology, Guangzhou 510006, China; yaocanzhong@scut.edu.cn

**Keywords:** EPU, transfer entropy, exchange rate

## Abstract

We investigate the strength and direction of information flow among economic policy uncertainty (EPU), US imports and exports to China, and the CNY/US exchange rate by using the novel concept of effective transfer entropy (ETE) with a sliding window methodology. We verify that this new method can capture dynamic orders effectively by validating them with the linear transfer entropy (TE) and Granger causality methods. Analysis shows that since 2016, US economic policy has contributed substantially to China-US bilateral trade and that China is making passive adjustments based on this trade volume. Unlike trade market conditions, China’s economic policy has significantly influenced the exchange rate fluctuation since 2016, which has, in turn, affected US economic policy.

## 1. Introduction

Causality is a central concept in natural sciences and is commonly understood to describe a situation in which a process evolving in time has some observable effect on a second process. However, the nature of this causative effect is challenging to describe and quantify with precision. There is a long history of determining whether some change truly causes another [1,2], especially if the effect is not deterministic and is observed only in aggregate.

The Granger causality test, which was first proposed in 1969 [3], is a statistical hypothesis test for determining whether one time series is useful for forecasting another. In the simplest terms, the so-called Granger causality describes the extent to which a response in the dependent series can be explained by a change in the first.

Transfer entropy arises from the formulation of conditional mutual information; when conditioning on the past values of variables, it quantifies the reduction in uncertainty provided by these past values when predicting the dependent variable, which presents a natural way to model the statistical causality between variables in multivariate distributions. In the general formulation, transfer entropy is a model-free statistic that can measure the time-directed transfer of information between stochastic variables and therefore provides an asymmetric method to measure information transfer.

As presented in this paper, transfer entropy appears naturally as a generalization of Granger causality. In fact, it has been shown that for multivariate normally distributed statistics, in which the relationship is therefore linear, this is indeed the case; Granger causality and transfer entropy are equivalent [4]. Granger causality and transfer entropy, which are both data-based causality analysis techniques, have been compared [4], and some of their advantages and disadvantages have been described. Duan et al. [5] demonstrated and compared different techniques for the root cause diagnosis of plant-wide oscillations. They found that Granger causality is relatively easy to implement, robust to data selection, and has a low computational burden and that its application techniques are well developed. However, it is suitable only for linear relationships between variables and may be prone to model misspecification.

Lindner et al. [6] presented a comparative analysis of Granger causality and transfer entropy when used for fault diagnosis in an industrial process. They found transfer entropy to be more generalizable and visually interpretable and Granger causality to be easier to automate, to be much less computationally expensive and to obtain easier-to-interpret values.

Though developed relatively recently, information theoretic methods have been used with success in research across disciplines to detect information transfer when interventionist approaches are not possible.

The information transfer method has been widely used in the finance field. This approach was employed by Kwon and Yang [7] to measure the relationship between equity indices, showing that the information transfer was greatest from the US and towards the Asia Pacific region. In particular, the S&P500 was shown to be the strongest driver of other stock indices. In an earlier and somewhat related work, Marschinski and Kantz [8] defined and used effective transfer entropy to quantify contagion in financial markets. Similarly, Tungsong et al. [9] developed the previous work by Diebold and Yilmaz [10] in quantifying spillover effects between financial markets, generalizing the methodology and estimating the time evolution of interconnectedness between financial systems. Kyrtsou, C et al. [11] introduced a Granger causality test using the nonlinear statistic of asymmetric partial transfer entropy to explore the complex relationships between the S&P500, VIX and volume. Dimpfl and Peter [12] proposed an appropriate bootstrap to derive confidence bounds and showed by means of a simulation study that standard linear approaches in economics and finance, such as vector autoregressions and Granger causality tests, are not well suited to detect information transfer. Garcia-Medina et al. [13] used random matrix theory and information theory to analyze the correlations and flow of information between 64,939 news items from The New York Times and 40 world financial indices during 10 months during the 2015–2016 period. Their results suggested a deep relationship between news and world indices and illustrated a situation in which news drives world market movement.

Compared to real variables, economic policy uncertainty (EPU) data are more forward-looking and therefore may be more relevant for determining the extent of a country’s economic influence on others. Previous studies have investigated the influence of EPU on a country’s economy. Han et al. [14] found that the US EPU significantly affects economic growth in China. Fontaine et al. [15] investigated the effects of China’s EPU on US macroeconomic activity using a nonlinear VAR model. They demonstrated that Chinese EPU significantly affects several US economic variables, though only during a recession. Li and Peng [16] found that the US EPU is a significant factor affecting stock market comovements between China and the US. Liow et al. [17] examined the international spillovers of the EPU of seven economies and financial market distress and showed that EPU leads this relationship. Hammoudeh et al. [18] investigated the impact of the US EPU on the US, European, Asian and Islamic stock markets and reported that uncertainty shocks explain an important share of changes in all four stock markets. It has been widely recognized that EPU has a significant impact on stock markets [19,20,21,22,23,24,25,26] and bond markets [27]. Other relevant studies have focused on the dependence between EPU and monetary policies [28], unemployment rates [29], exchange rates [30] and exchange rate expectations [31].

Motivated by these previous studies, we follow the thinking regarding the information flow and causal relationship to study the interaction among EPU, trade and the exchange rate. The rest of this paper is organized as follows. In the next section, we introduce the traditional effective transfer entropy (ETE) method. In Section 3, we propose an improved ETE method based on sliding windows and compare it with the linear TE and Granger causality methods. In Section 4, we describe the time series data used in the models. In Section 5, we present the empirical results obtained with the new model and analyze an alternative mechanism. Finally, we provide the conclusions of this paper.

## 2. Methodology

### 2.1. Linear Causality

We model a time series as autoregressive by expressing its value *Y_t_* at time *t* as a sum of the contributions over *m* distinct lagged series using the following linear equation:

(1) $$Y_{t}=\sum_{k=1}^{m}\beta_{k}^{\left(Y\right)}Y_{t-k}+\varepsilon_{t}$$

where $\beta_{k}^{\left(Y\right)}$ is a general coefficient term and $\varepsilon_{t}$ is the residual. Linear regression estimates the coefficient parameters $\beta_{k}^{\left(Y\right)}$, which minimize the sum of squared residuals. To detect whether the values of some second time series X anticipate the future values of Y, we can compare Equation (1) with the following:

(2) $$Y_{t}=\overset{m}{\underset{k=1}{\sum }}\beta_{k}^{\prime \left(Y\right)}Y_{t-k}+\overset{m}{\underset{k=1}{\sum }}\beta_{k}^{\prime \left(X\right)}X_{t-k}+\varepsilon_{t}^{\prime }$$

We determine that the distribution Y is Granger caused by X if the residual in the second regression is significantly smaller than the residual in the first regression. When this distribution holds, then there must be some information transfer from X to Y. Following Geweke [32], we can represent the information transfer by

(3) $$TE_{X\to Y}=\frac{1}{2}\log\left(\frac{var\left(\varepsilon_{t}\right)}{var\left(\varepsilon_{t}^{\prime }\right)}\right)$$

where we adopt the transfer entropy notation (TE), following Barnett et al. [4], which shows Granger causality to be equivalent to transfer entropy for multivariate normal distributions.

### 2.2. Nonlinear Causality

To detect nonlinear causality, we apply an information-theoretic approach. Equation (3) measures the extent to which the additional information in the lagged variable reduces the variance in the model residuals. Transfer entropy extends this concept by considering the uncertainty instead of the variance. Adopting Shannon’s measure of information [33], we can express the uncertainty associated with the random variable X by

(4) $$H\left(X\right)=-\underset{x\in X}{\sum }p\left(x\right)\log p\left(x\right)$$

where H(x) is termed the Shannon entropy of the distribution and p(x) represents the probability of X=x. If the system states can be described by two subsystems X and Y, we can use the joint entropy $H\left(X,Y\right)=-\underset{x\in X}{\sum }\underset{y\in Y}{\sum }p\left(x,y\right)\log p\left(x,y\right)$ to describe the state of the system. If two systems interact with each other and we know the outcome of one of them, then it may decrease the unpredictability of the other.

(5) $$H\left(Y|X\right)=-\underset{x\in X}{\sum }p\left(x\right)\underset{y\in Y}{\sum }p\left(y|x\right)\log p\left(y|x\right)=-\underset{x,y}{\sum }p\left(x,y\right)\log\frac{p\left(x,y\right)}{p\left(x\right)}$$

where H(Y|X) is called the conditional entropy, which is the uncertainty of Y such that X is known, i.e.,

(6) H(Y|X)=H(X,Y)−H(X)

Another necessary concept to be defined is the mutual information (MI), indicating the mutual dependence between X and Y and defined as

(7) $$MI\left(X;Y\right)=-\underset{x,y}{\sum }p\left(x,y\right)\log\frac{p\left(x,y\right)}{p\left(x\right)p\left(y\right)}$$

When two random variables share information, the mutual information is given by

(8) MI(X;Y)=MI(Y;X)=H(Y)−H(Y|X)=H(X)−H(X|Y)

The entropy of Y conditioned on two variables is

(9) H(Y|X,Z)=H(X,Y,Z)−H(X,Z)

and the conditional mutual information is therefore

(10) MI(X;Y|Z)=H(Y|Z)−H(Y|X,Z)

Now, for each lag k, we can describe the information transfer from $X_{t-k}$ to $Y_{t}$ in terms of the following conditional mutual information:

(11) $$TE_{X\to Y}^{\left(k\right)}=MI\left(Y_{t};X_{t-k}|Y_{t-k}\right)=H\left(Y_{t}|Y_{t-k}\right)-H\left(Y_{t}|X_{t-k},Y_{t-k}\right)$$

This equation represents the resolution of uncertainty in predicting Y when considering the past values of both Y and X compared with considering the past values of Y alone.

Considering Equations (6) and (9), we can therefore represent the transfer entropy for a single lag k, which is shown in Equation (11), in terms of four separate joint entropy terms. Following Equation (4), these terms may be estimated with the data using a nonparametric density estimation of the probability distributions. For multivariate normal statistics, Equations (3) and (11) coincide [4].

### 2.3. Effective Transfer Entropy (ETE)

It is a feature of the nonparametric estimation of entropy that the absolute scale of the transfer entropy measure has only limited meaning; to detect causality, a relative position must be considered. A simple technique proposed by Marschinski and Kantz [8] is the ETE, derived by subtracting from the observed transfer entropy an average transfer entropy figure calculated over independently shuffled time series, which destroys the temporal order and hence any possible causality. We adopt a shuffling approach producing 50 null-hypothesis transfer entropy values from independently shuffled time series over the same domain containing no causality. By calculating the mean and standard deviation of the shuffled transfer entropy figures, we estimate the significance of a causal result as the distance between the result and the average shuffled result, standardizing by the shuffled standard deviation:

(12) $$ETE=TE-{\bar{TE}}_{shuffle}$$

where ${\bar{TE}}_{shuffle}$ is the mean of the shuffled values and $\sigma_{shuffle}$ is the standard deviation. The shuffling of the time series destroys temporality and should ensure that the mean is approximately zero; therefore, the spread of the data dictates the significance of the result.

(13) $$Z:=\frac{TE-{\bar{TE}}_{shuffle}}{\sigma_{shuffle}}$$

Assuming that the distribution is close to Gaussian, we can say that a result with Z>3 is roughly in the top 1% of the results, and hence is comparable to a *p*-value of 0.01. The nature of the method typically enables clearer significance to be observed for fewer shuffles, even without a strictly Gaussian distribution, so it is computationally more attractive than the *p*-value.

This expression corresponds to the degree to which the result lies in the right tail of the distribution of the zero-causality shuffled samples and hence how unlikely the result is to be due to chance. Therefore, the *Z*-score figure represents the significance of the excess transfer entropy in the unshuffled case. We compute the *Z*-score in Equation (13) for both linear and nonlinear results.

## 3. An Improved Effective Transfer Entropy Method Based on a Sliding Window 

### 3.1. Improved Method Based on a Sliding Window and Comparison with the Traditional Linear Method

A previous study has shown that the nonlinear TE method is useful for detecting nonlinear processes [34]. However, the lag order these authors found was global and unique and was not suitable for capturing the accurate order between two nonstationary series. For nonstationary time series, the dependency structure between the explanatory variables and explained variables evolves dynamically. Due to policy or unexpected events, the causal structure between real financial sequences tends to change over time. Therefore, it would be inaccurate if a single lag were used to measure the global causality. In addition, the other major problem with traditional linear TE is its inability to identify short-term memory correlation features in time series-dependent structures.

In fact, most financial time series have memory characteristics. The fluctuation in price at one time point will cause persistent fluctuation at the next time point, and this memory will also be transferred to another time series, resulting in a correlation with memory features. Using a single lag order cannot reflect the memory of the lead-lag order. Based on the sliding window method, during the sliding process, one window and the other window basically overlap for a large part of the data before completely nonintersecting. This overlap also lasts a long time, so it can reflect certain memory characteristics.

Considering that the locality of nonstationary data may be stationary or approximately stationary and can characterize the memory features well, this paper proposes an improved transfer entropy method based on a sliding window. The improved method calculates the transfer entropy in the same way as described in Section 2.2, but is limited to a certain time segment. Through forward scrolling, the transfer entropy at each time point is obtained, and the causal relationship between the two times series is revealed. In addition to being able to capture the structural changes between two time series, the improved method can also help trace back the specific time period of the structural change, which cannot be done based on the traditional linear TE method. We next verify the validity of the algorithm.

We can generate a time series X following the geometric Brownian motion according to Equation (14):

(14) $$X_{t+1}=\left(1+\mu \right)X_{t}+\sigma X_{t}\eta_{t}$$

where $\eta_{t}$ is noise obeying the standard normal distribution, $\eta_{t}∼N\left(0,1\right)$, and μ and σ represent the drift coefficient and the diffusion coefficient, respectively. Y depends on X, and the equation is constructed as follows (Equation (15)):

(15) $$Y_{t}=\left(1-\alpha \right)X_{t-k}+\alpha X_{t-k}^{\prime }$$

where $X_{t-k}^{\prime }$ is another time series generated according to Equation (14). k is the given lag order, and α∈(0,1) determines the dependence strength between the series Y and X.

Assuming k=2,α=0.5;k=4,α=0.5;k=5,α=0.5, we can obtain a time series with length 200, i.e.,$X_{t},Y_{t}^{k=2};X_{t},Y_{t}^{k=4}$ and $X_{t},Y_{t}^{k=5}$ according to Equations (14) and (15).

**Figure 1:**
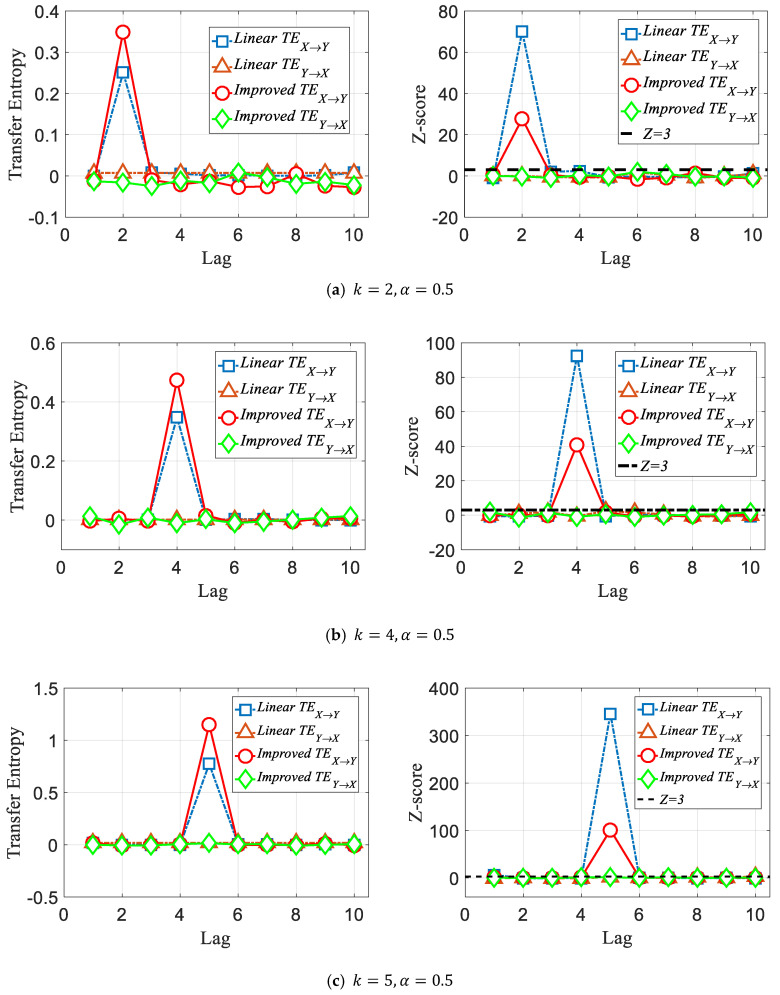
Demonstration that both methods identify the true lag values with maximal transfer entropy. Nonlinear transfer entropy is calculated using a quantile-binned histogram, with 6 classes per dimension, over 2500 points. The *Z*-score for each result is also plotted for both methods. According to the Z>3 principle, it can be concluded that for two time series with a single lag order, both methods can accurately identify the lag orders.

**Figure 2:**
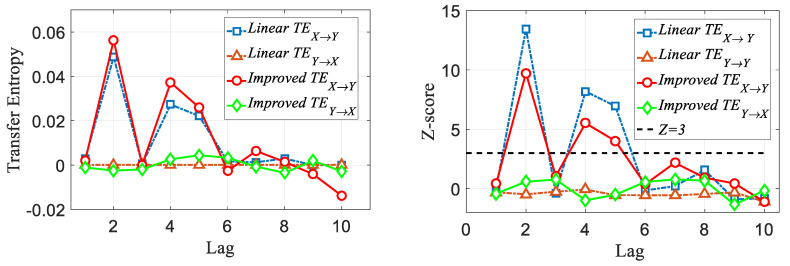
Demonstration that both methods identify the true lag values with maximal transfer entropy. The linear TE can only capture k=2, corresponding to the highest transfer entropy value (*Z*-score indicates the value is above a significant level), while the improved TE method can detect k=2, 4 and 5.

**Figure 3:**
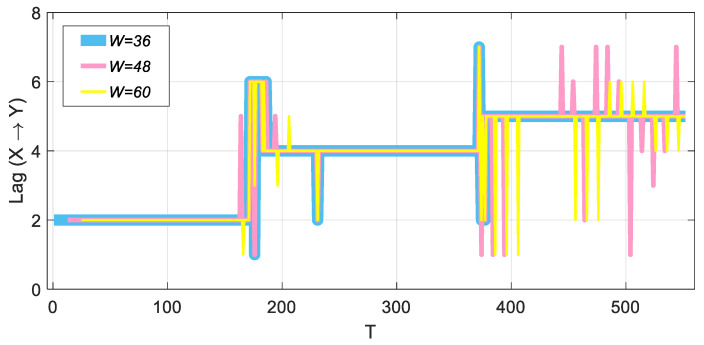
Comparison of improved TE method results based on different window lengths.

As shown in Figure 1, for a correlation series with a single lag structure, both the traditional transfer entropy, i.e., the linear TE and improved TE methods, can capture the lag order accurately. However, according to the *Z*-score significance test, we can see that when the temporal order is destroyed, the linear TE does not show significance in the relevant order; in other words, the linear TE method is dependent on the time evolution. As shown in Figure 2, the linear TE can identify only the order k=4, which is the highest corresponding transfer entropy value (the *Z*-score indicates that the value is above a significant level). However, the improved TE could detect both k=4 and k=5. Moreover, as shown in Figure 3, we can also track the specific time period when the lead–lag order fluctuates with the improved method.

We reshape $[X_{t},\;Y_{t}^{k=2}],\;[X_{t},Y_{t}^{k=4}]$ and $\left[X_{t},\;Y_{t}^{k=5}\right]$ into two new time series $X_{t}^{\prime },\;Y_{t}^{\prime }$, where $X_{t}^{\prime }=\left[X_{t},X_{t},X_{t}\right]$ and $Y_{t}^{\prime }=\left[Y_{t}^{k=2},Y_{t}^{k=4},Y_{t}^{k=5}\right]$. These new series show obvious structural fluctuations, and the features are more in line with the characteristics of real financial data.

Due to the shortcomings of traditional linear methods in revealing dynamic orders, in the empirical analysis in Section 5, we apply the improved transfer entropy to explore the information flow between all sequences. The sliding window length of all of these structures is 36 months with a forward step size of 1 month.

### 3.2. Sliding Window Length

When the length is too large, the sliding window easily covers the breakpoints when the structure changes so that it cannot serve the purpose of capturing the structural changes; when the window length is too small, the amount of grouped data is too small for the kernel density estimation, i.e., the degree of freedom is insufficient, which affects the estimation effect. Therefore, the window value of the sliding window may affect the analysis results, so we next compare three different lengths.

Figure 3 shows the performance of the improved method under different window lengths. It can be seen from the figure that when W=48 and W=60, a large fluctuation occurs for the order k=5. However, overall, the jump performance of the order is very obvious, and the improved *TE* can still accurately identify three different orders, although there are fluctuations when identifying higher orders.

### 3.3. Comparison with the Granger Causality Test

Granger causality is a regression-based interpretation of Wiener’s causality definition [35]. It is essentially a test of whether a lagging variable of one variable can be introduced into an equation containing other variables. If a variable is affected by the lag of other variables, they are considered to have Granger causality. For the sequences X and Y, using different lag orders, we can obtain the causality test results for the two sequences (Table 1). In this section, the Granger causality test is employed as a comparison with the improved TE to detect the true lag orders. Referring to Granger’s work [3], we model the Granger causality test with the following two regression equations:

(16) $$X_{t}=\overset{p}{\underset{i=1}{\sum }}\alpha_{i}X_{t-i}+u_{t}$$

(17) $$X_{t}=\overset{p}{\underset{i=1}{\sum }}\rho_{i}X_{t-i}+\overset{p}{\underset{i=1}{\sum }}c_{i}Y_{t-i}+v_{t}$$

where X denotes the object needed to find the Granger cause, Y denotes the object needed to determine whether it can Granger cause X, and residuals *u_t_* and *v_t_* are assumed to be mutually independent and individually distributed with a zero mean and constant variance. These equations were tested using the following hypothesis:

**H0.**  Y *does not Granger cause* $X\left(c_{1}=c_{2}=\ldots =c_{p}=0\right)$ *.*

*F*-test can be expressed as follows:

(18) $$F=\frac{\left(RSS_{0}-RSS_{1}\right)/p}{RSS_{1}/\left(n-2p-1\right)}∼F\left(p,n-2p-1\right)$$

where ${RSS}_{0}$ is the residual sum of squares of Equation (16), ${RSS}_{1}$ is the residual sum of squares of Equation (17), n is the number of observations, and p is a lag value. We reject hypothesis H0 and accept that Y is a Granger cause of X if and only if F>F(p,n−2p−1). The model order p can be determined by minimizing AIC [36], defined as

(19) $$AIC\left(p\right)=2\log\left(\left|\sigma \right|\right)+\frac{2m^{2}p}{n}$$

where σ is the estimated noise covariance, m is the dimension of the stochastic process and n is the length of the data window used to estimate the model. For example, to detect the causal relationship from exports to the US EPU, Y should be set to the export sequence, while X should be set to the US EPU sequence. Conversely, Y should be set to the US EPU before detecting the causal relationship from the US EPU to exports.

The Granger causality test based on the sliding window method can also obtain the order and significance of two series’ correlations. Taking Y and X as an example, we elaborate the processes of the Granger model estimation within a fixed window as follows:

(1) The maximum of the lag value *p* is set to a fixed number, such as 10.
(2) Calculating the total *AIC* of Equations (16) and (17) by traversing the *p*-value from 1 to 10, we obtain the corresponding *p* of a minimum AIC. The experimental results show that the optimal p is 5.
(3) Equations (16) and (17) are estimated using OLS with p=5.
(4) F and F(p,n−2p−1) are calculated according to Equation (18). The results show F=4.0635 and F(p,n−2p−1)=3.8549 (at the 99% confidence level).
(5) If F>F(p,n−2p−1), we conclude that Y can Granger cause X significantly.
(6) The window is moved forward by a one-month step, and steps (1)–(5) are repeated.

**Figure 4:**
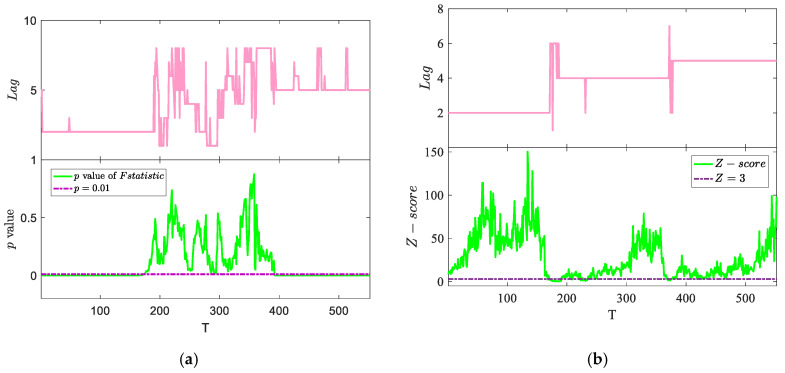
Comparison of the Granger causality method based on sliding window and the improved TE method. The widow length of both methods W=36, and the significance level is 1%. (**a**) Granger causality test based on the sliding window method. *p*-value <1% means that the causal relationship is significant in this area. The Granger method can clearly identify k=2, but it cannot identify k=4, and there is considerable noise interference when identifying k=5. (**b**) The result based on the improved TE method. Z>3 indicates a significance level of *p*-value <1% (Section 3.3). The improved TE method can clearly identify the three orders 2, 4, and 5.

Using the process described above, we obtained the Granger causality test results based on window length W=36 (Figure 4a). As shown in Figure 4a, although the Granger causality test can identify k=2, it cannot effectively capture the two orders of 4 and 5. For k=4, it cannot pass the significance test; for k=5, although it can pass the significance test, there may be other orders, such as k=8. The improved TE method can accurately identify three different orders (Figure 4b); in addition, the stage where the order jumps cannot pass the significance test.

The improved method based on the sliding window performs better than the traditional linear TE, which is specifically reflected in the following: (1) Not only can it identify the global single lag order, but for the system with dynamic structure changes, it can better capture multiple lead-lag orders, and identify the turning time points. (2) The improved algorithm can identify short-term memory correlation features. It is worth noting that the improved algorithm is not suitable for causal sequences of long-term memory features. In fact, theoretical TE-related methods are suitable for analyzing long-term memory-dependent structure systems. For this type of system, it is best to use the thermal optimization path method or multifractal analysis method [37].

## 4. Data Description

**Figure 5:**
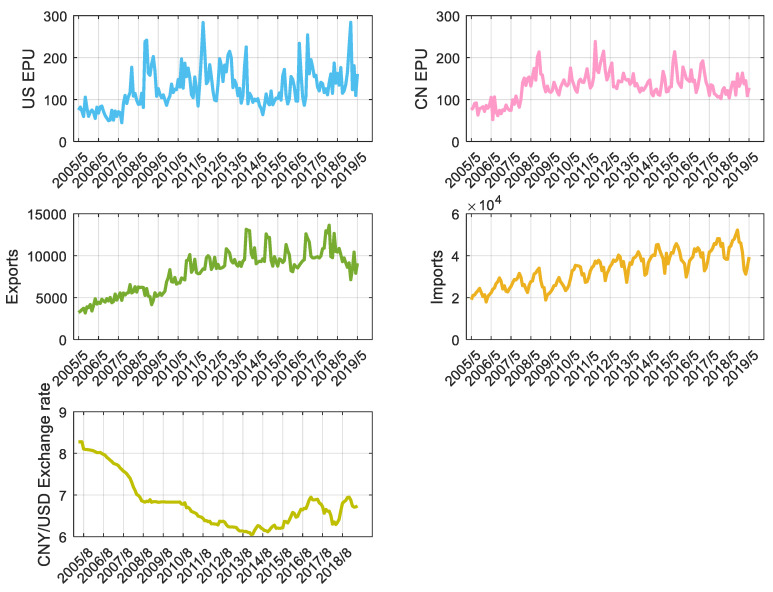
Time series of the US EPU index, the CN EPU index, US imports from China, US exports to China and the CNY/USD exchange rate.

The export and import data on US trade in goods with China are collected from the United States Census Bureau (Figure 5b,c). All figures are in millions of US dollars on a nominal basis and are not seasonally adjusted unless otherwise specified. Detailed numbers may not equal the total due to rounding. The range of the export and import data covers May 2005 to May 2019.

Baker et al.’s [38] index of EPU (Figure 5a) is available from the economic policy uncertainty website (http://www.policyuncertainty.com/). The EPU index for the US relies on 10 leading newspapers: USA Today, the Miami Herald, the Chicago Tribune, the Washington Post, the Los Angeles Times, Boston Globe, the San Francisco Chronicle, the Dallas Morning News, the New York Times, and the Wall Street Journal. Baker et al. [38] searched the digital archives of each paper that contain the following terms: ‘uncertainty’ or ‘uncertain’; ‘economic’ or ‘economy’; and one of the following policy terms: ‘congress,’ ‘deficit,’ ‘Federal Reserve,’ ‘legislation,’ ‘regulation’ or ‘White House’. The article targeted must contain terms in all three categories pertaining to uncertainty, the economy, and policy. EPU indices are created by checking whether the article also contains one or more category-specific policy terms, as listed in Table 2. Detailed calculations can be found in the reference [38] and http://www.policyuncertainty.com/.

The China economic uncertainty index used in this article was compiled by Paul Luk’s research team [39] at Hong Kong Baptist University. They constructed the index using search words from ten Hong Kong newspapers: Wen Wei Po, Sing Pao, etc. For each newspaper, the research group counted articles that contain Chinese words related to economic uncertainty and policy to formulate the economic policy uncertainty index. The most recent data from the index and related papers can be downloaded from the web: https://economicpolicyuncertaintyinchina.weebly.com/. The range of the US EPU and the CN EPU is from May 2005 to May 2019.

CNY/USD (Figure 5d) was collected from the website: https://excelrates.com/historical-exchange-rates/USD-CNY. The range of the series is from April 2005 to May 2019.

The descriptive statistics are shown in Table 3. According to Jarque-Bera statistics, we can determine that the US EPU and exchange rate series exhibits high peaks and fat tails. Therefore, it is inappropriate to analyze the time series directly using the transfer entropy or the Granger causality method. In the following transfer entropy analysis, we use the logarithmic difference form of the time series.

## 5. Empirical Results

Since traditional linear methods cannot identify the dynamic orders between time series and unable to track specific time points when structural fluctuations occur, we apply the improved transfer entropy to explore the information flow between all sequences.

### 5.1. EPU and Exports/Imports

**Figure 6:**
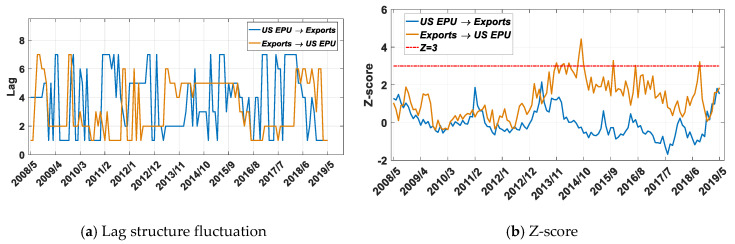

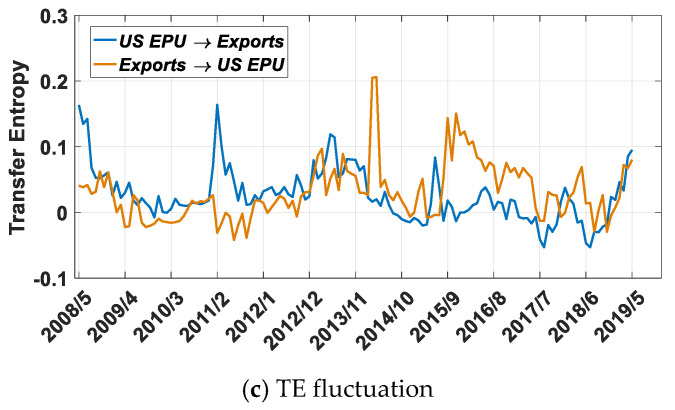
Results for transfer entropy between the US EPU and exports based on the improved TE method. The time period is from 1 May 2005 to 1 May 2019, and the sliding window length is 36 months, with a 1-month step size.

Based on the improved TE method, we analyze the information flow between EPU and US-China imports and exports. As shown in Figure 6a, there is an obvious dynamic order fluctuation in the correlation between EPU and US exports to China. As shown in Figure 6b, there are almost no regions with a *Z*-score higher than 3, which indicates that the US EPU has no significant impact on US exports to China.

Figure 6c shows that before August 2013, the EPU’s transfer entropy to exports was higher than the impact of exports on EPU, but after 2013, the situation changed, and the impact of exports on EPU gradually exceeded the impact of EPU on exports. This result may be related to China’s emergence as the third largest exporter to the US in 2013, and the huge Chinese market affects the formulation of US economic policies. China is the fastest-growing export market for the US. In 2013, US exports to China reached USD 120 billion, second only to the US’s two trading partners in the North American Free Trade Zone, Canada and Mexico, making China the third largest exporter to the US. Our conclusions verify that since 2013, the US’s huge export trade to China has become an important engine for US economic growth, and US economic policy has begun to pay more attention to the issue of China’s export trade.

**Figure 7:**
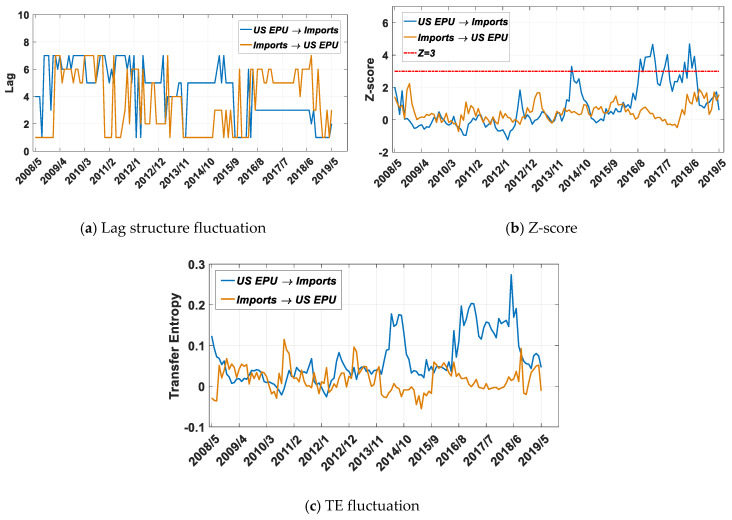
Results for transfer entropy between the US EPU and imports based on the improved Table 2005. to 1 May 2019, and the sliding window length is 36 months, with a 1-month step size.

As the significance test for EPU and import volume shows (Figure 7b), after 2016, which basically covers the period of the Trump administration in the US (Donald Trump officially took office in 2017), EPU has had a significant impact on import volume. This stage also represents a period of high uncertainty in US trade policy. It can also be seen from Figure 7c that since 2016, there have been significant asymmetric characteristics between the impact of EPU on imports and that of imports on EPU. Therefore, EPU can be used to explain and predict the fluctuation of imports from China, which has become an important source of imports. Referring to Figure 7a, it can be found that the EPU leading order is 3 months.

From Figure 5, we can see that the uncertainty in US economic policy has not increased abnormally since 2016. There is no significant difference from the fluctuation before 2016, but combined with information flow analysis, economic policy uncertainty has a greater impact from a local perspective. When discussing the value added or employment of the national import industry, the US should seriously consider the impact of policy uncertainty.

Combined with the results shown in Figure 6, it can be concluded that the US EPU has no significant impact on US exports to China. However, since the Trump administration took office in 2016, the uncertainty of US policy has had a significant impact on imports from China with a 3-month lag order, and the effect is also characterized by periodic fluctuations.

**Figure 8:**
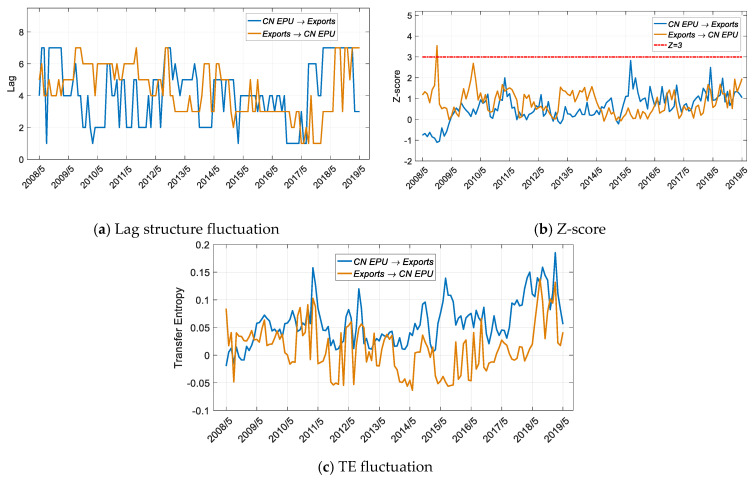
Results for transfer entropy between the CN EPU and exports based on the improved Table 2005. to 1 May 2019, and the sliding window length is 36 months, with a 1-month step size.

As shown in Figure 8, China’s economic policy does not affect the volume of US exports to China. In contrast, according to the results shown in Figure 7, US imports from China impact China’s economic policies. The significant impact of the US EPU on imports from China occurred in 2010 with a lead of 2 months, and after 2019, with a lead of 3 months.

**Figure 9:**
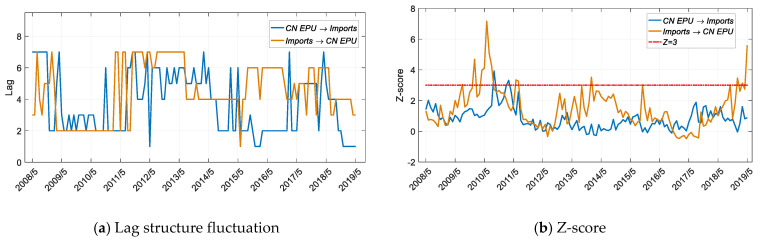

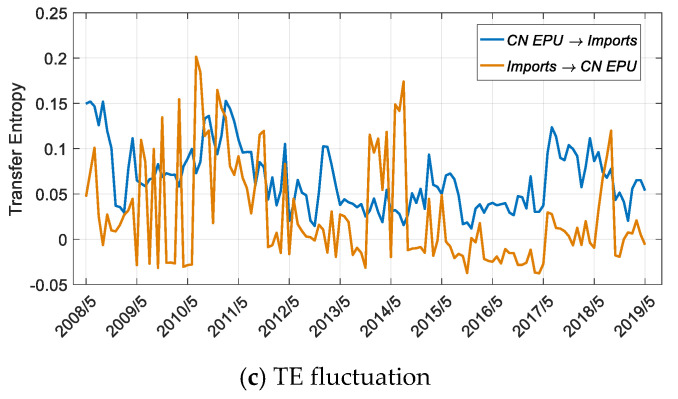
Results for transfer entropy between the CN EPU and imports based on the improved TE method. The time period is from 1 May 2005 to 1 May 2019, and the sliding window length is 36 months, with a 1-month step size.

China’s economic policy does not affect exports from the US to China, nor does it affect US imports from China, indicating that China’s economic policies do not have a substantial impact on trade. In contrast, since 2016, US economic policies have had an impact on imports from China (Figure 7b), causing China’s economic policies to be adjusted accordingly (Figure 9b). US economic policy has been the main factor affecting bilateral trade since 2016, and China is making passive adjustments based on this trade volume.

In the literature [40], Zhang et al. showed that the US EPU Granger caused that of China except during recent years and around the 2008 global financial crisis. As a result, they suggested that investors conducting business in China should pay more attention to the important role played by US economic uncertainty and its impact on that of China. Our results validate this view. In fact, when we introduce the intermediate variable of trade volume, we revealed that the spillover effect of the US EPU on the CN EPU occurs mainly through import trade from China.

### 5.2. EPU and Exchange Rate

Theoretically, EPU has both direct and indirect impacts on exchange rate volatility. However, there is a scarce body of literature on the impact of EPU on exchange rate volatility ([41,42]). If there is a completely free trade market between countries, then there is a certain internal relationship between the exchange rate and trade. However, when a trade war occurs, the policies formulated by each country may directly affect the trade volume or the exchange rate. At this time, the relationship between the trade market and the exchange rate market is more complicated, and it is often difficult to maintain the correlation of previous fluctuations.

**Figure 10:**
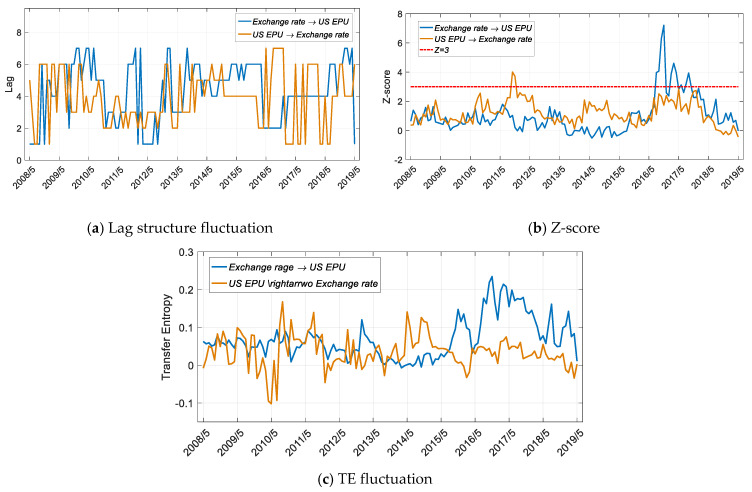
Results for transfer entropy between the US EPU and the exchange rate based on the improved TE method. The time period is from 1 May 2005 to 1 May 2019, and the sliding window length is 36 months, with a 1-month step size.

**Figure 11:**
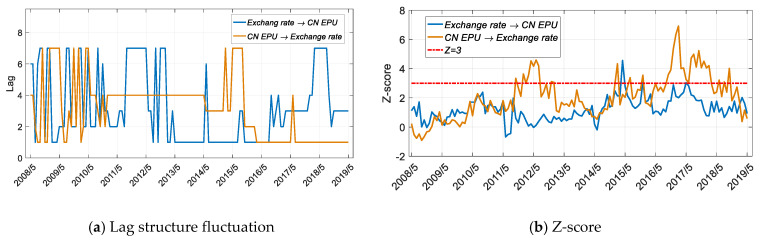

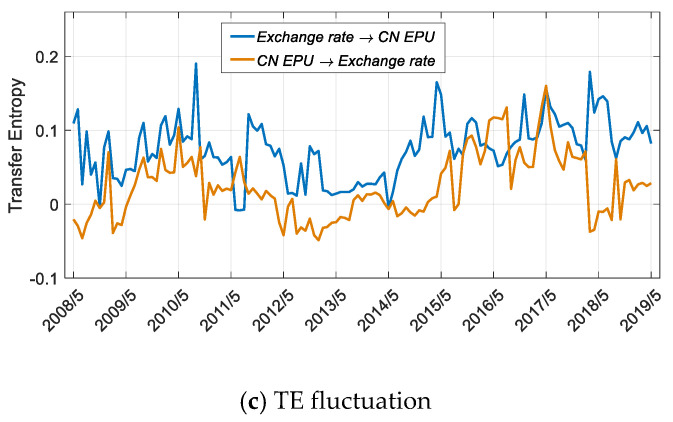
Results for transfer entropy between the CN EPU and the exchange rate based on the improved TE method. The time period is from 1 May 2005 to 1 May 2019, and the sliding window length is 36 months, with a 1-month step size.

As shown in Figure 10b and Figure 11b, since 2016, China and the United States have a significant entropy relationship with the exchange rate, but the direction of information flow is different.

It can be seen from Figure 10b that beginning in June 2016, the impact of the exchange rate on US policy is significant. After May 2017, this effect tends to be nonsignificant, but the impact of the exchange rate on policy is still higher than the impact of policy on the exchange rate at this stage (Figure 10c). It can be concluded from Figure 10c that in most other periods of time, the US EPU and the exchange rate do not have a significant relationship. This means that, first, the exchange rate is actually relatively independent and not affected by government economic policies. Moreover, since the Trump administration took office and the new government came to power in 2016, US economic policy has undergone major changes, as reflected in greater fluctuations in CNY/US exchange rates.

In the literature [42], Chen et al. concluded that there was an asymmetric impact of EPU on exchange rate volatility in China, and this analysis result was also verified in Figure 11b,c. However, [42] did not identify the specific time period for the asymmetric correlation between the policy and the exchange rate market, nor did it analyze the difference in the impact of China–US policy uncertainty on exchange rate fluctuations. As can be seen from Figure 11b, China’s economic policy had a significant impact on the exchange rate beginning in June 2016, with a leading order of one month (Figure 11a), and this influence tended to be nonsignificant after May 2018. In addition, in 2012, China’s economic policy had a significant impact on the exchange rate.

Therefore, from the perspective of the entropy analysis, China’s economic policy will affect the exchange rate market, which is related to China’s implementation of a noncompletely floating exchange rate system. Since 2016, China’s economic policy has caused significant changes in the exchange rate, which has, in turn, affected US economic policy, which has subsequently caused the influence of China’s policy changes to be nonsignificant. This process reflects the complexity of the mutual linkage of economic policies between the two countries. Unlike trade market conditions, China’s economic policy did not directly affect bilateral trade, but it affected US policy through the exchange rate market.

## 6. Conclusions

The ETE method can identify only a single lag order and cannot trace the specific time when the impact of the event fluctuates. This article first proposes an improved ETE method with a sliding window and proves the effectiveness of the algorithm. Compared with the linear method, the improved TE method can capture all the lag orders more accurately, especially when the order changes frequently. In addition, the improved TE method outperforms the Granger causality test in identifying the lag orders. When the information flow relationship between two sequences is time-varying, Granger’s method cannot identify certain orders or has large noise.

Based on the correlation analysis between EPU and US imports and exports to China, the analysis shows that the US EPU has no significant impact on US exports to China. However, since the Trump administration took office in 2016, the uncertainty of US policy has had a significant impact on imports from China with a 3-month lag order, and the effect is also characterized by periodic fluctuations. China’s economic policy does not affect the volume of US exports to China; however, US imports from China will have an impact on China’s economic policies. A significant impact of the US EPU on imports from China occurred in 2010 with a lead of 2 months and after 2019, with a lead of 3 months. Since 2016, US economic policy has contributed substantially to China–US bilateral trade, and China is making passive adjustments based on this trade volume.

Unlike trade market conditions, China’s economic policy has not directly affected bilateral trade, but it has affected US policy through the exchange rate market. China’s economic policy affects the exchange rate market, which is related to China’s implementation of a noncompletely floating exchange rate system. Since 2016, China’s economic policy has significantly caused exchange rate change, which, in turn, has affected US economic policy, causing the effects of China’s policy changes to become nonsignificant once again.

The results of our analysis indicate this phenomenon. Since 2016, the friction between China and the United States in the field of trade has been increasing. In this Sino–US trade war, the United States has more frequently resorted to tariff policies to affect imports, while China prefers to use exchange rate instruments. The measures on both sides have caused the other party’s countermeasures, as reflected in the significant lag in economic policies.

## Figures and Tables

**Table 1:** Granger causality test results.

Lag	1	2	3	4	5	6	7	8	9	10
*F*	0.5664	4.0797	2.9568	2.2895	6.6669	5.8331	4.9915	5.0310	4.4082	3.9662
*p*-value	0.4520	0.0174	0.0319	0.0586	4.76 × 10^−6^ *	6.48 × 10^−6^ *	1.68 × 10^−5^ *	4.75 × 10^−6^ *	1.38 × 10^−5^ *	3 × 10^−5^ *

Note: * *p*-value <0.01 means that the test result rejects the null hypothesis significantly, and at least one lag variable X Granger causes Y. Therefore, the orders in which X Granger causes Y are 6, 7, 8, 9 and 10.

**Table 2:** Category-specific policy term sets of EPU indices.

Category-Specific Policy Term Sets	Related Terms	1985:1-2014:12 Overall Average
Economic Policy Uncertainty		100.0
Fiscal Policy	Anything covered by Taxes or Government Spending & Other	46.1
-Taxes	Taxes, tax, taxation, taxed	40.3
-Government Spending & Other	Government spending, federal budget, budget battle, balanced budget, defense spending, military spending, entitlement spending, fiscal stimulus, budget deficit, federal debt, national debt, etc.	17.1
Monetary Policy	Federal reserve, the Fed, money supply, open market operations, quantitative easing, monetary policy, Fed funds rate, overnight lending rate, Bernanke, Volcker, central bank, interest rates, Fed chairman, Fed chair, discount window, European Central Bank, ECB, etc.	28.1
Healthcare	Health care, Medicaid, Medicare, health insurance, malpractice tort reform, malpractice reform, prescription drugs, drug policy, food and drug administration, etc.	17.3
National Security	National security, war, military conflict, terrorism, terror, 9/11, defense spending, military spending, police action, armed forces, base closure, military procurement, etc.	23.8
Regulation	Anything covered by financial regulation and truth in lending, union rights, card check, collective bargaining law, national labor relations board, minimum wage, living wage, right to work, closed shop, etc.	17.4
-Financial Regulation	Banking (or bank) supervision, Glass-Steagall, tarp, thrift supervision, Dodd-frank, financial reform, commodity futures trading commission, CFTC, house financial services committee, Volcker rule, etc.	3.3
Sovereign Debt & Currency Crises	Sovereign debt, currency crisis, currency devaluation, currency revaluation, euro crisis, Eurozone 51 crisis, exchange rate, European debt, Asian financial crisis, Russian crisis, etc.	1.6
Entitlement Programs	Entitlement program, entitlement spending, government entitlements, social security, Medicaid, government welfare, welfare reform, unemployment insurance, unemployment benefits, food stamps, EITC, etc.	12.4
Trade Policy	Import tariffs, import duty, import barrier, government subsidies, government subsidy, WTO, World Trade Organization, trade treaty, trade agreement, trade policy, etc.	3.8

**Table 3:** Descriptive statistics for the series.

	IM	EX	CN EPU	US EPU	Exchange Rate CNY/USD
**Count**	173	173	173	173	170
**Mean**	33,273.0705	7938.5202	130.4408	124.9687	6.8104
**Std**	8143.0326	2582.7937	33.8313	48.2120	0.6087
**Min**	16,184.9000	2609.3000	52.1958	44.7828	6.0540
**Max**	52,202.3000	13,630.3000	238.3172	284.1359	8.2765
**Skewness**	−0.0292	−0.1197	0.1333	0.8279	0.9972
**Kurtosis**	−0.8894	−0.7930	0.2282	0.5363	−0.0514
Jarque-Bera	5.7268	4.9460	0.8876	21.837055 *	28.1910 *

Note: * indicates that the *p*-value of the Jarque-Bera statistic is less than 0.01, which can be considered as rejecting the null hypothesis of the normal distribution.
